# A commentary on ‘Circulating metabolic markers after surgery identify patients at risk for severe postoperative complications: a prospective cohort study in colorectal cancers’

**DOI:** 10.1097/JS9.0000000000001547

**Published:** 2024-05-03

**Authors:** Yajie Wang, Huan Deng, Yisheng Pan

**Affiliations:** Department of Gastrointestinal Surgery, Peking University First Hospital, Beijing, People’s Republic of China

*Dear Editor,*

We were interested to read the impressive study by Montcusí *et al*.^1^ in *International Journal of Surgery* and would like to congratulate the authors for their interesting findings. This study provides new insights into the relationship between circulating metabolites and susceptibility to severe complications after colorectal cancer (CRC) by analyzing the peripheral blood metabolomics of 150 colorectal patients in a single center before and after surgery. The study found that surgery-induced metabolic changes were associated with the severity of short-term postoperative complications. They found changes in 119 metabolic markers after CRC surgery, especially phosphatidylcholines (PCs), lysophosphatidylcholines (LPCs), sphingomyelins (SMs), acylcarnitines, and branched-chain amino acids (BCAAs), which shows that the preoperative metabolome is not associated with severe postoperative complications, suggesting that metabolites associated with postoperative prognosis are primarily influenced by surgical presentation. These findings have the potential to help identify patients at high risk for serious complications and early decision-making. However, we wish to discuss the following concerns.

First of all, this observational study adopts single-center research, which has the advantages of being controllable and convenient. The data collection and patient management are highly consistent. This certainly adds a lot of credibility to the findings. However, the data of single-center studies are only from a single medical institution or research center, and the results may be limited by the specific characteristics and patient population of the center, which often has shortcomings such as small sample size and large selection bias. They may also be influenced by the center’s specific environment and operational procedures, such as the degree to which surgical procedures are standardized and postoperative management, which may affect the reliability and generality of results.

Secondly, this paper focuses on the postoperative complications caused by anastomotic leakage (AL). So, the existence of an anastomosis is a very important question. In CRC surgery, one of the most important things is the mode of operation. Generally speaking, for colon cancer or rectal cancer more than 5 cm from the anus, prerectal resection (Dixon surgery) is performed^2^; that is, after the tumor is removed, the colorectal is anastomosed in the abdominal cavity. While for patients with low rectal cancer whose inferior margin of the tumor is located less than 5 cm from the anus, abdominal perineal combined rectal cancer resection (Miles surgery) is usually performed^3^, that is, distal closure, and permanent transperitoneal resection of the sigmoid colon. In this case, there is no intestinal anastomosis, so there is no anastomotic leakage. Therefore, we suggest that the author specify the surgical methods performed by the patients in the study, and stratify the discussion between the patients who underwent Miles surgery and those who underwent Dixon surgery or take the surgical methods as a covariable, which will make the final results more convincing.

Finally, we truly thank the authors for their excellent and important work.

## Ethical approval

Because this is a commentary of a research paper, ethical approval is not required.

## Consent

Because this is a commentary of a research paper, consent is not required.

## Sources of funding

This project was supported by the National Natural Science Foundation of China (81970459).

## Author contribution

Y.W.: literature collection and manuscript writing; H.D.: manuscript revised; Y.P.: project development.

## Conflicts of interest disclosure

All authors declare that they do not have any conflicts of interest.

## Research registration unique identifying number (UIN)

Because this is a commentary on a research paper, Research Registration is not required.

## Guarantor

Yisheng Pan.

## Data availability statement

This manuscript is a comment. All the data from the current study are publicly available.

## Provenance and peer review

Our paper was not invited.
